# Supplementary material for: Electrochemical Immunosensors with PQQ-Decorated Carbon Nanotubes as Signal Labels for Electrocatalytic Oxidation of Tris(2-carboxyethyl)phosphine
Source: Nanomaterials (Basel). 2021 Jul 5;11(7):1757. doi: 10.3390/nano11071757 (PMC8308108; doi:10.3390/nano11071757)
Supplement: Supplementary file 1 [file nanomaterials-11-01757-s001.zip › nanomaterials-1273578-supplementary.pdf]

Electrochemical immunosensor with PQQ-decorated carbon nanotube as signal label for electrocatalytic oxidation of tris(2-carboxyethyl)phosphine

Xiaohua Ma[1], Dehua Deng[2], Ning Xia[2], Yuanqiang Hao[1,*], Lin Liu[1,2,*]

[1] Henan Key Laboratory of Biomolecular Recognition and Sensing, Shangqiu Normal University, Shangqiu, Henan 476000, People's Republic of China

[2] College of Chemistry and Chemical Engineering, Anyang Normal University, Anyang, Henan 455000, People's Republic of China

[*]Corresponding authors: haoyuanqiang@aliyun.com (Y.H.); liulin@aynu.edu.cn (L.L.)

Results

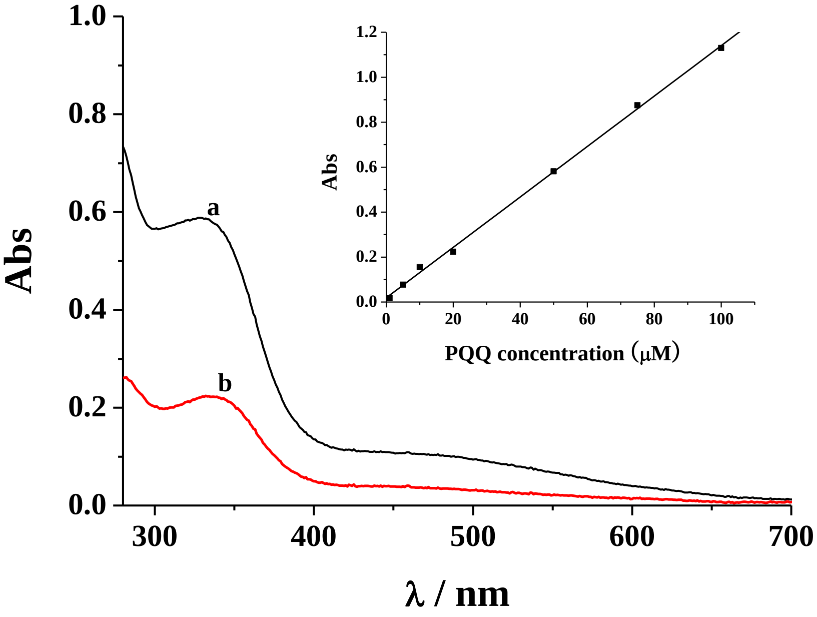

Fig. S1 UV-Vis spectra of the PQQ/EDC mixed solution before (curve a) and after (curve b) incubation with CNT-NH_2 for 12 h. Before the measurement, the suspension was centrifugated at 14000 rpm for 10 min and the resulting supernatant solution was diluted 50 times with DMF. The inset shows the plot of the adsorption intensity of PQQ at the concentration of 1, 5, 10, 20, 50, 75 and 100 μM.

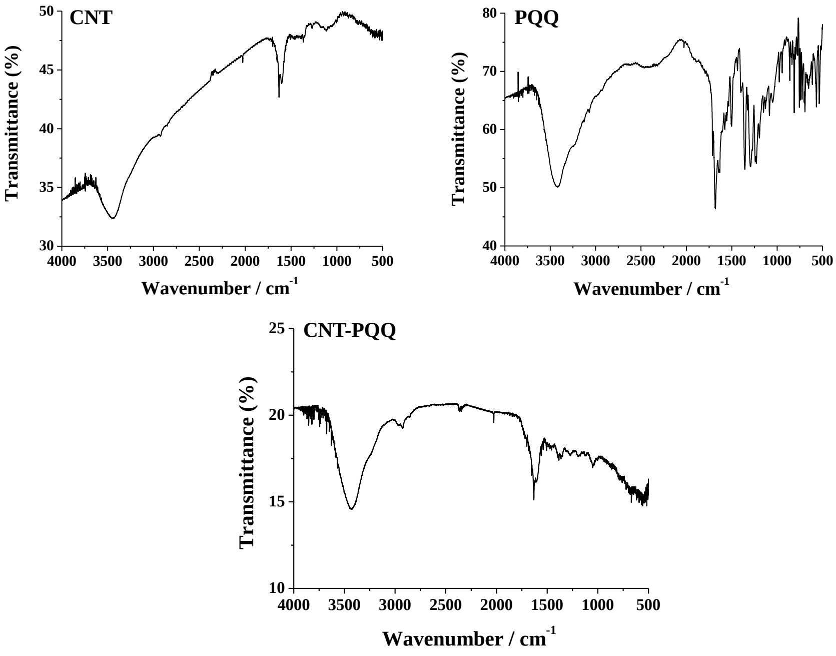

Fig. S2 Fourier transform infrared (FTIR) spectroscopy of CNT, PQQ and CNT-PQQ.

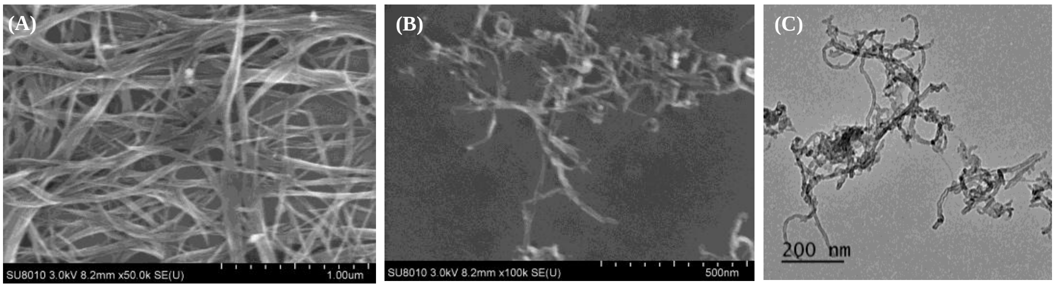

Fig. S3 SEM images of the untreated CNT-NH_2 (A) and the resulting PQQ-CNT-Ab_2 (B). Panel C shows the TEM image of PQQ-CNT-Ab_2.

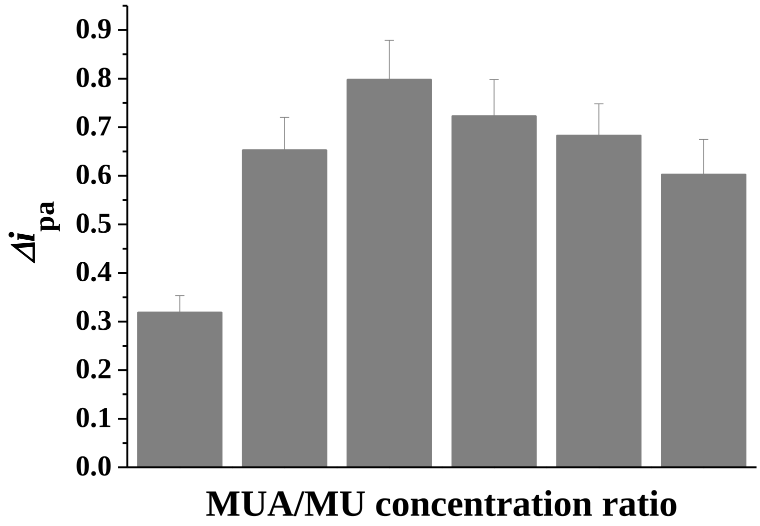

Fig. S4 Effect of the MUA/MU ratio on Δi_{pa}. The concentrations of PSA, FcM and TCEP were 10 ng/mL, 50 μM and 200 μM, respectively. The scan rate was 20 mV/s.

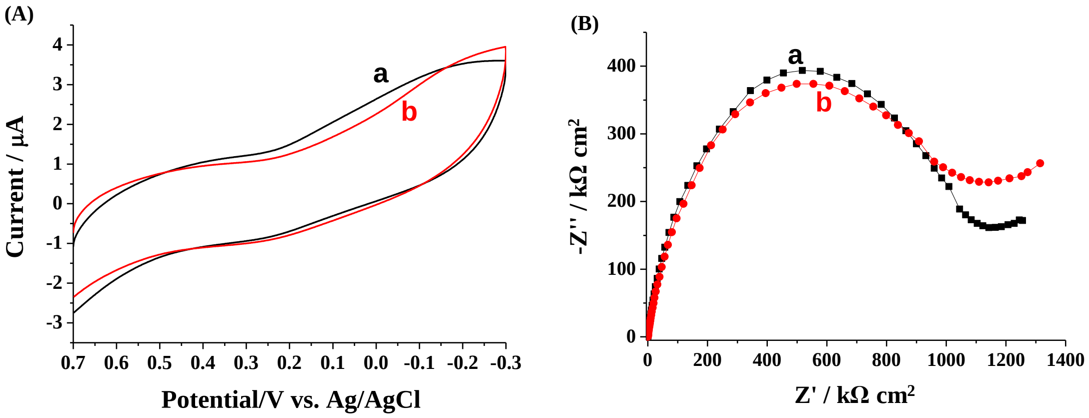

Fig. S5. CVs (A) and EIS (B) of $[Fe(CN)_6]^{3-}$ at the sensor electrode before (curve a) and after (curve b) capture of PSA.

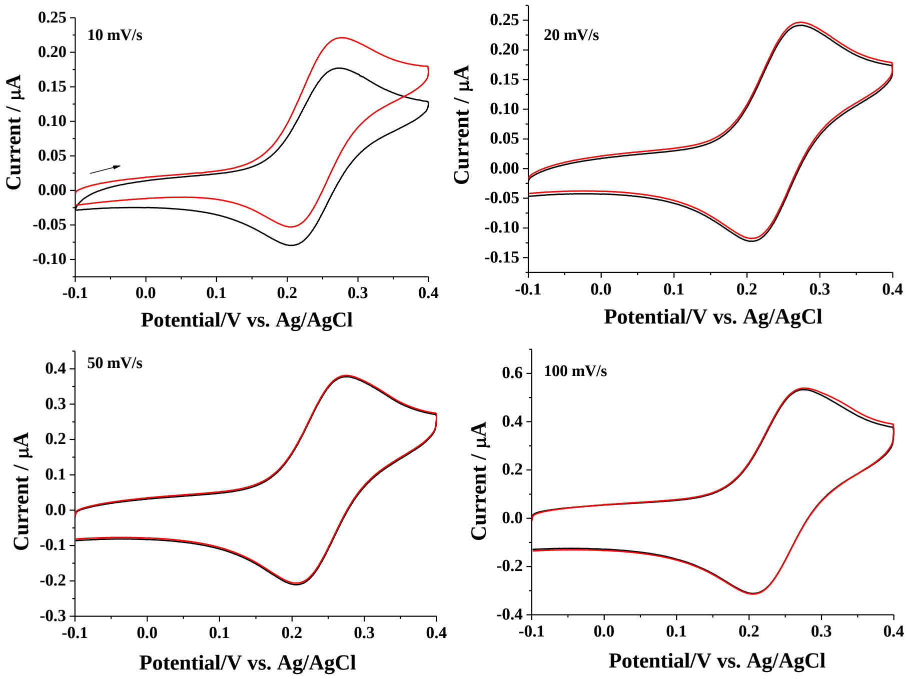

Fig. S6 CVs of 50 μM FcM in the absence (black curve) and presence (red curve) of 200 μM TCEP at a scan rate of 10, 20, 50 or 100 mV/s.

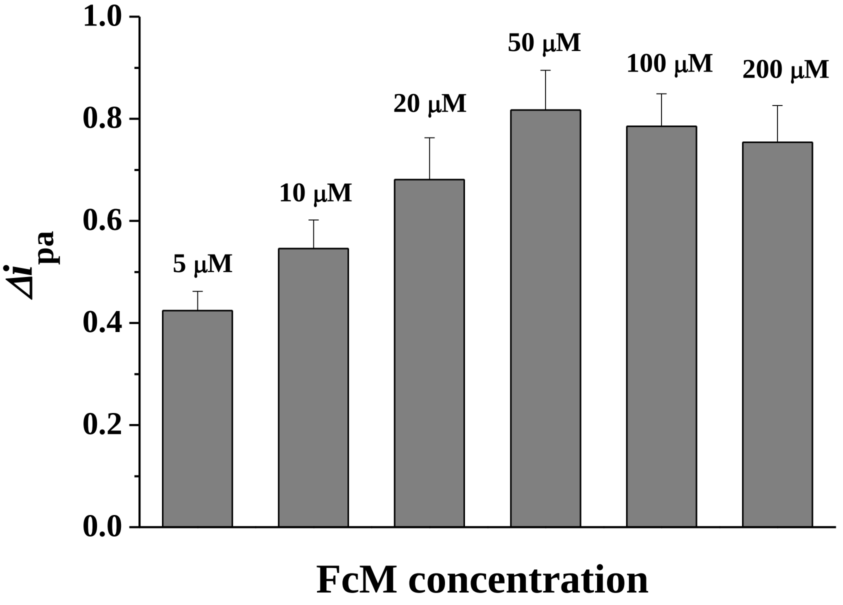

Fig. S7 Dependence of Δi_{pa} on FcM concentration. The concentrations of PSA and TCEP were 10 ng/mL and 200 μM, respectively.

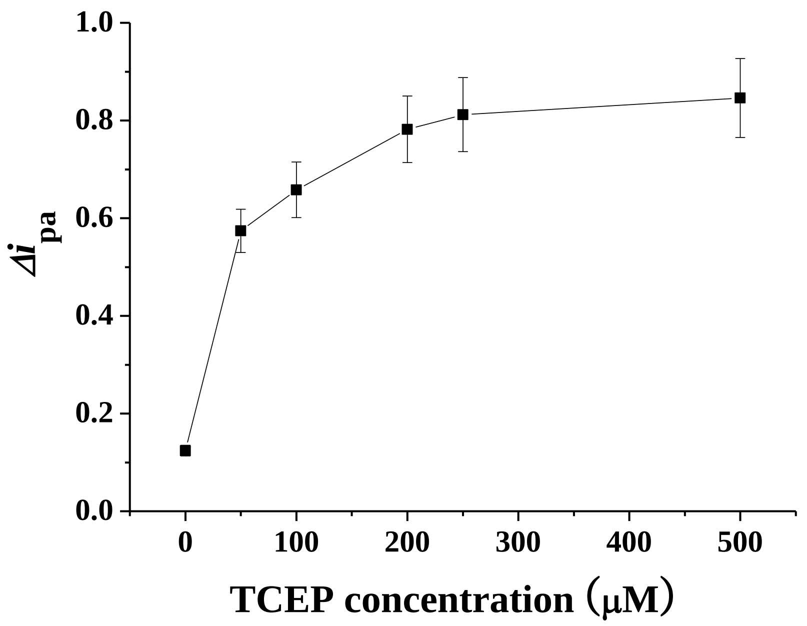

Fig. S8 Dependence of Δi_{pa} on TCEP concentration. The concentrations of PSA and FcM were 10 ng/mL and 50 μM, respectively.

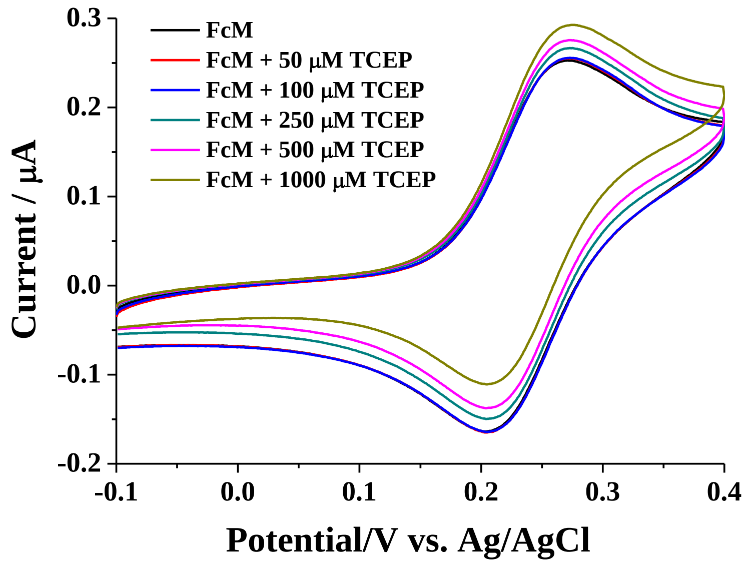

Fig. S9 CVs of 50 μM FcM in the presence of different concentrations of TCEP at a scan rate of 20 mV/s.

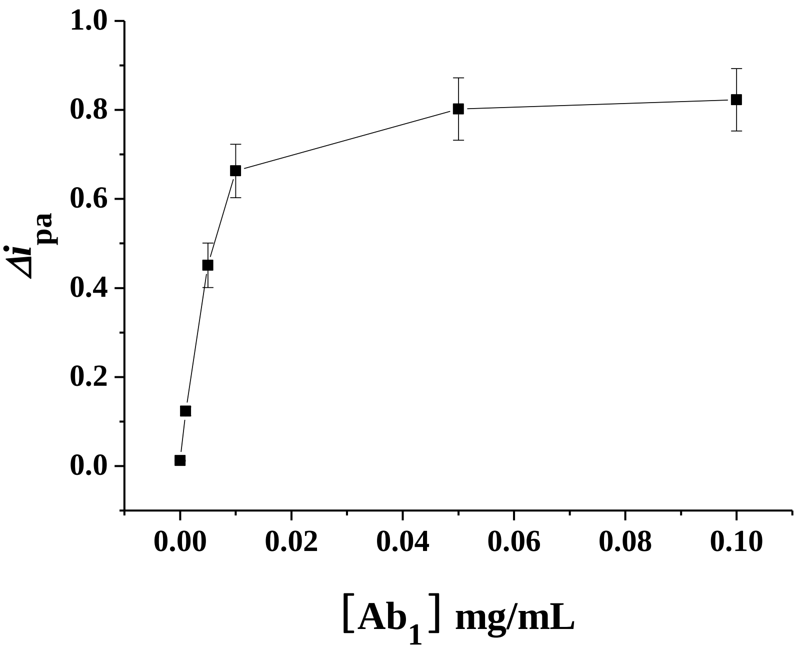

Fig. S10 Dependence of Δi_{pa} on Ab_1 concentration. The concentrations of PSA, FcM and TCEP were 10 ng/mL, 50 μM and 250 μM, respectively. The scan rate was 20 mV/s.

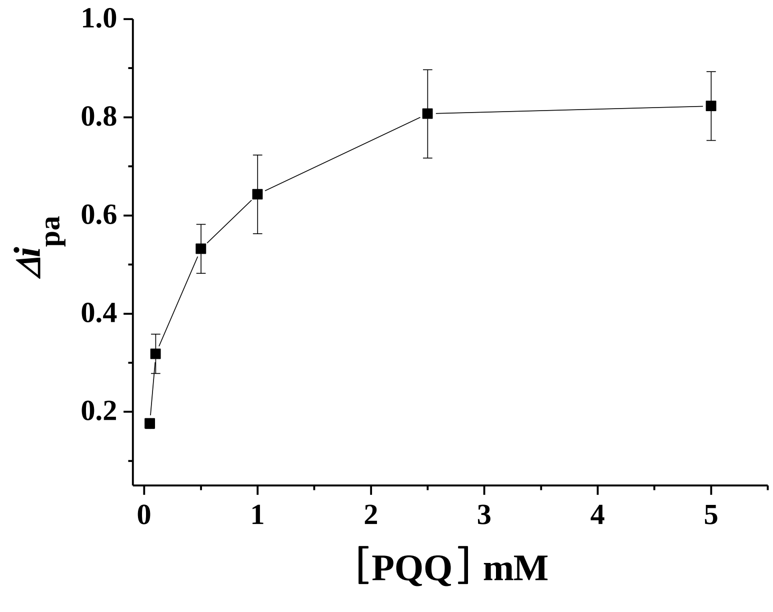

Fig. S11 Dependence of Δi_{pa} on PQQ concentration used for the preparation of PQQ-CNT. The concentrations of PSA, FcM and TCEP were 10 ng/mL, 50 μM and 250 μM, respectively. The scan rate was 20 mV/s.

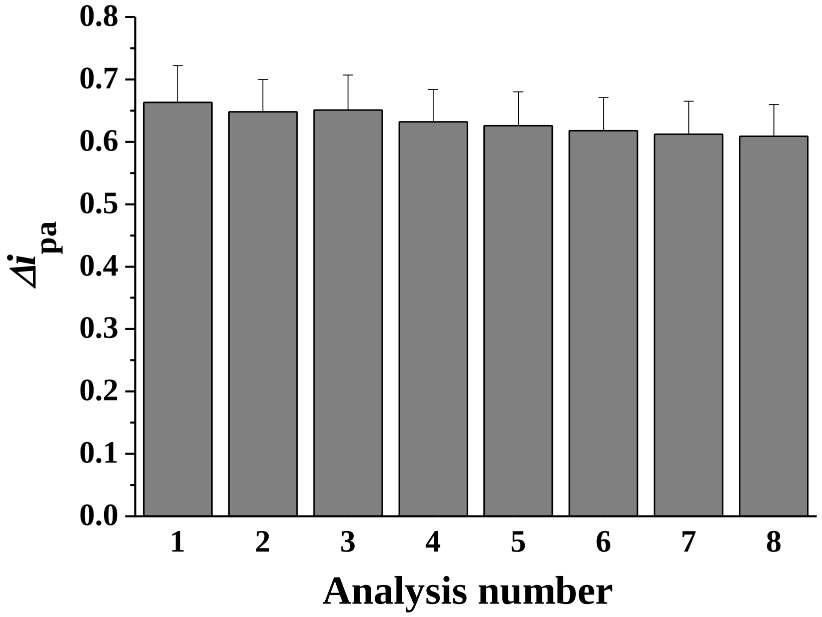

Fig. S12 Dependence of Δi_{pa} on the analysis/regeneration number. The other experimental conditions are the same as those of Fig. S11.

Table S1. Analytical performances of sandwich-type electrochemical immunosensors with different nanocatalysts as signal labels.

Electrode modifier	target	Label	substrate	LOD (pg/mL)	Linear range (ng/mL)	Ref.
Au-GS	PSA	rGO-MWCNT-Pd	H_2O_2	0.17	0.0005–15	[1]
GS	AFP	Au-Pd	H_2O_2	5	0.05–30	[2]
BSA-AuNPs	BTB	MSC-PdNS	Thi/H_2O_2	5	0.01–10	[3]
GS	CEA	Fe_3O_4@MnO_2@Pt	H_2O_2	0.16	0.0005–20	[4]
graphene	CEA	M-Pt NPs	H_2O_2	7	0.02–20	[5]
PDA-N-MWCNT	AFP	GS/Au@Pt	H_2O_2	0.05	0.1–10	[6]
N-GS	SCCA	Pt-Fe_3O_4	H_2O_2	15.3	0.05–18	[7]
CD-NGs	CEA	NiAuPt-NGs	H_2O_2	0.27	0.001–100	[8]
graphene	DES	Pt@SBA-15	H_2O_2	0.28	0.001–10	[9]
	ES			1.2	0.005–8	
rGO-TEPA	TSGF	Ag@CeO_2	H_2O_2	0.2	0.0005–0.1	[10]
Au/chitosan	CEA	AgNCs/GRO	H_2O_2	0.037	0.0001–100	[11]
rGO-Au NPs	CEA	SWCNTs@GQDs	H_2O_2	5.3	0.05–0.65	[12]
Au@SH-GS	SCCA	Au/Ag/Au NPs	H_2O_2	0.18	0.5–40	[13]
CFGO	IgG	Cu@TiO_2	H_2O_2	0.052	0.0001–100	[14]
Au@APTES	PSA	Cu_2O	H_2O_2	0.05	0.00005–0.1	[15]
AuNPs	AFP	PDA	FDM	0.3	0.001–50	[16]
AuNF	PCT	Fc-Fc/β-CD/PAMAM-Au	AA	0.36	0.0018–500	[17]
AuNPs	PSA	Pd NPs/ Co-MOF	H_2O_2	0.03	0.0001–50	[18]
SAMs	PSA	PQQ-CNT	FcM/TCEP	5	0.005–1	This work

Abbreviations: GS, graphene sheets; rGO, reduced graphene oxide; MWCNT, multiwalled carbon, AFP, α-fetoprotein; AuNPs, Au nanoparticles; BTB, brevetoxin B; MSC-PdNS mesoporous carbon-enriched palladium nanostructure; Thi, thionine; CEA, carcinoembryonic antigen; M-Pt NPs, mesoporous platinum nanoparticles; N-MWCNT, N-doped MWCNT; N-GS, nitrogen-doped GS; NiAuPt-NGs, NiAuPt nanoparticles on graphene nanosheets; DES, diethylstilbestrol; ES, estradiol; Pt@SBA-15, platinum nanoparticle functionalized mesoporous silica nanoparticles; TEPA, tetraethylene pentamine; TSGF, tumor specific growth factor; AgNCs/GRO, silver nanoclusters and graphene oxide nanocomposite; GQDs, grapheme quantum dots; SH-GS, mercapto-functionalized GS; SCCA, squamous cell carcinoma antigen; CFGO, carboxyl functionalized graphene oxide; APTES, 3-aminopropyltriethoxysilane; AuNF, Au nanoflower; PDA, polydopamine; FDM, 1,1′-ferrocene dimethanol; PCT, procalcitonin; Fc-Fc, N,N-bis(ferrocenoyl)-diaminoethane; β-CD, β-cyclodextrins; PAMAM-Au, poly(amidoamine) dendrimer-encapsulated Au nanoparticles; NGs, β-CD functionalized reduced graphene oxide nanosheets; AA, ascorbic acid; GP-P3ABA, graphene-poly(3-aminobenzoic acid).

References

1. Tian, L.; Liu, L.; Li, Y.; Wei, Q.; Cao, W. 3D sandwich-type prostate specific antigen (PSA) immunosensor based on rGO–MWCNT–Pd nanocomposite. *New J. Chem.* **2015**, *39*, 5522.
2. Zhao, L.; Li, S.; He, J.; Tian, G.; Wei, Q.; Li, H. Enzyme-free electrochemical immunosensor configured with Au–Pd nanocrystals and N-doped graphene sheets for sensitive detection of AFP. *Biosens. Bioelectron.* **2013**, *49*, 222.
3. Lin, Y.; Zhou, Q.; Lin, Y.; Lu, M.; Tang, D. Mesoporous carbon-enriched palladium nanostructures with redox activity for enzyme-free electrochemical immunoassay of brevetoxin B. *Anal. Chim. Acta* **2015**, *887*, 67.
4. Wu, D.; Ma, H.; Zhang, Y.; Jia, H.; Yan, T.; Wei, Q. Corallite-like magnetic Fe_3O_4@MnO_2@Pt nanocomposites as multiple signal amplifiers for the detection of carcinoembryonic antigen. *ACS Appl. Mater. Interfaces* **2015**, *7*, 18786.
5. Cui, Z.; Wu, D.; Zhang, Y.; Ma, H.; Li, H.; Du, B.; Wei, Q.; Ju, H. Ultrasensitive electrochemical immunosensors for multiplexed determination using mesoporous platinum nanoparticles as nonenzymatic labels. *Anal. Chim. Acta* **2014**, *807*, 44.
6. Jiao, L.; Mu, Z.; Zhu, C.; Wei, Q.; Li, H.; Du, D.; Lin, Y. Graphene loaded bimetallic Au@Pt nanodendrites enhancingultrasensitive electrochemical immunoassay of AFP. *Sensor. Actuat. B: Chem.* **2016**, *231*, 513.
7. Wu, D.; Fan, H.; Li, Y.; Zhang, Y.; Liang, H.; Wei, Q. Ultrasensitive electrochemical immunoassay for squamous cell carcinoma antigen using dumbbell-like Pt-Fe3O4 nanoparticles as signal amplification. *Biosens. Bioelectron.* **2013**, *46*, 91.
8. Tian, L.; Liu, L.; Li, Y.; Wei, Q.; Cao, W. Ultrasensitive sandwich-type electrochemical immunosensor based on trimetallic nanocomposite signal amplification strategy for the ultrasensitive detection of CEA. *Sci. Rep.* **2016**, *6*, 30849.
9. Ma, H.; Mao, K.; Li, H.; Wu, D.; Zhang, Y.; Du, B.; Wei, Q. Ultrasensitive multiplexed immunosensors for the simultaneous determination of endocrine disrupting compounds using Pt@SBA-15 as a non-enzymatic label. *J. Mater. Chem. B* **2013**, *1*, 5137.
10. Yu, S.; Zou, G.; Wei, Q. Ultrasensitive electrochemical immunosensor for quantitative detection of tumor specific growth factor by using Ag@CeO2 nanocomposite as labels. *Talanta* **2016**, *156-157*, 11.
11. Wang, J.; Wang, X.; Wu, S.; Song, J.; Zhao, Y.; Ge, Y.; Meng, C. Fabrication of highly catalytic silver nanoclusters/graphene oxide nanocomposite as nanotag for sensitive

electrochemical immunoassay. *Anal. Chim. Acta* **2016**, *906*, 80.
12. Luo, Y.; Wang, Y.; Yan, H.; Wu, Y.; Zhu, C.; Du, D.; Lin, Y. SWCNTs@GQDs composites as nanocarriers for enzyme-free dualsignal amplification electrochemical immunoassay of cancer biomarker. *Anal. Chim. Acta* **2018**, *1042*, 44.
13. Wang, Y.; Zhang, Y.; Su, Y.; Li, F.; Ma, H.; Li, H.; Du, B.; Wei, Q. Ultrasensitive non-mediator electrochemical immunosensors using Au/Ag/Aucore/double shell nanoparticles as enzyme-mimetic labels. *Talanta* **2014**, *124*, 60.
14. Zhang, S.; Ma, H.; Yan, L.; Cao, W.; Yan, T.; Wei, Q.; Du, B. Copper-doped titanium dioxide nanoparticles as dual-functional labels for fabrication of electrochemical immunosensors. *Biosens. Bioelectron.* **2014**, *59*, 335.
15. Ma, H.; Li, Y.; Wang, Y.; Hu, L.; Zhang, Y.; Fan, D.; Yan, T.; Wei, Q. Cubic Cu_2O nanoframes with a unique edgetruncated structure and a good electrocatalytic activity for immunosensor application. *Biosens. Bioelectron.* **2016**, *78*, 167.
16. Xiang, H.; Wang, Y. u.; Wang, M.; Shao, Y.; Jiao, Y.; Zhu, Y. A redox cycling-amplified electrochemical immunosensor for α-fetoprotein sensitive detection via polydopamine nanolabels. *Nanoscale* **2018**, *10*, 13572.
17. Shen, W.-J.; Zhuo, Y.; Chai, Y.-Q.; Yang, Z.-H.; Han, J.; Yuan, R. Enzyme-free electrochemical immunosensor based on host-guest nanonets catalyzing amplification for procalcitonin detection. *ACS Appl. Mater. Interfaces* **2015**, *7*, 4127.
18. Dai, L.; Li, Y.; Wang, Y.; Luo, X.; Wei, D.; Feng, R.; Yan, T.; Ren, X.; Du, B.; Wei, Q. A prostate-specific antigen electrochemical immunosensor based on Pd NPs functionalized electroactive Co-MOF signal amplification strategy. *Biosens. Bioelectron.* **2019**, *132*, 97.
